# Targeting the PLK1-FOXO1 pathway as a novel therapeutic approach for treating advanced prostate cancer

**DOI:** 10.1038/s41598-020-69338-8

**Published:** 2020-07-23

**Authors:** Lilia Gheghiani, Shengzhe Shang, Zheng Fu

**Affiliations:** Department of Human and Molecular Genetics, VCU Institute of Molecular Medicine, VCU Massey Cancer Center, School of Medicine, Virginia Commonwealth University, Richmond, VA 23298 USA

**Keywords:** Targeted therapies, Prostate cancer, Oncogenes

## Abstract

The forkhead box protein O1 (FOXO1) is considered to be a key tumor suppressor due to its involvement in a broad range of cancer-related functions, including cellular differentiation, apoptosis, cell cycle arrest, and DNA damage. Given that inactivation of FOXO1 has been reported in many types of human cancer, we sought to investigate whether restoration of the pro-apoptotic activity of FOXO1 may be used as a new promising strategy for cancer treatment. Our previous study revealed that Polo-like kinase 1 (PLK1), a serine/threonine kinase that is essential for cell cycle progression, is a novel and major regulator of FOXO1 in the late phases of the cell cycle. Here, we provided evidence that PLK1-dependent phosphorylation of FOXO1 induces its nuclear exclusion and negatively regulates FOXO1′s transcriptional activity in prostate cancer (PCa). Blocking the PLK1-dependant phosphorylation of FOXO1 restored the pro-apoptotic function of FOXO1 in PCa. Combining PLK1 inhibition with nocodazole (to induce mitotic arrest) had synergistic antitumor effects in vitro, with minimal effect on normal prostate epithelial cells. These findings shed light on a novel approach to reactivate apoptotic pathways in advanced PCa and support targeting PLK1-FOXO1 pathways as a novel approach for treating advanced PCa.

## Introduction

Prostate cancer (PCa) is one of the most frequently diagnosed neoplasms in Western countries and the second leading cause of cancer-related death in American men^1^. Despite improved diagnosis and early treatment (surgery and anti-androgen therapies), effective treatment for PCa remains a major unmet medical need, which relies upon the ability to elucidate the intricate relationships that govern cellular proliferation, metabolism, and survival.

Many human cancers are caused by the dysregulation of activity of transcription factors. Targeting transcription factors are among the few current promising strategies for cancer therapy^2^. Accumulating evidence highlights the forkhead box O (FOXO) family as a class of transcription factors that may serve as an important target for cancer therapy^3^. The FOXO proteins, including FOXO1, FOXO3a, FOXO4, and FOXO6, are implicated in a broad range of cancer-related functions, including cellular differentiation, apoptosis, cell cycle arrest, and DNA damage and repair^4^. FOXO1 is the most abundant protein of the family. Activation of FOXO1 leads to upregulation of pro-apoptotic genes, such as Bim, Fas ligand (FasL), and legumain^5–7^. In addition, FOXO1 regulates G_1_ and G_2_ cell cycle progression by modulating the expression of the cyclin-dependent kinase inhibitors p27^KIP1^ and p21^WAF1^, the retinoblastoma protein-related protein p130, cyclin D1 and D2, and GADD45^8–13^. FOXO1 also plays a role in the surveillance of DNA damage by transcriptionally regulating expression of DNA-damage response genes^4^. Interestingly, deletion of FOXO1 in mice facilitated prostate tumorigenesis^14^. These findings suggest that FOXO1 possesses tumor suppressor functions. The importance of FOXO proteins in tumor suppression is further supported by the fact that the *FOXO1*, *FOXO3a,* and *FOXO4* genes are all affected by chromosomal translocations detected in solid tumors and leukemia. Along the same lines, several reports demonstrated that activation of FOXO1 induces apoptosis in PCa cells^10,15,16^, suggesting that inhibition of FOXO1 function is critical for the survival of PCa cells and thereby has the potential to be exploited for targeted therapy for patients with PCa.

The transcriptional activity of FOXO1 is mainly regulated by its nuclear-cytoplasmic shuttling and primarily promoted by post-translational modifications, including phosphorylation, acetylation, and ubiquitination^17^. Our previous studies have shown that the serine/threonine kinase Polo-like kinase 1 (PLK1), an essential cell cycle regulator, is a major regulator of FOXO1^18^. FOXO1 negatively regulates the late phases of cell cycle progression^19^. PLK1 binds to and phosphorylates FOXO1 during the late phase of the cell cycle. This phosphorylation event induced the nuclear exclusion of FOXO1 and, consequently, led to the inhibition of FOXO1′s transcriptional activity in the late phases of the cell cycle^18^. Importantly, we reported that blocking PLK1-dependant phosphorylation of FOXO1 delays G_2_/M transition and promotes the activation of pro-apoptotic signaling pathways, leading to cell death^18^.

In this study, we set out to investigate the potential involvement of the PLK1-FOXO1 pathway in human PCa and to explore the therapeutic potential of this regulation. We show that PLK1-mediated phosphorylation of FOXO1 induces its nuclear exclusion, leading to the inhibition of FOXO1′s nuclear transcriptional activity in PCa cells. Furthermore, combining PLK1 inhibition with nocodazole had synergistic antitumor effects in vitro, with minimal effect on normal prostate epithelial cells. Therefore, our results provide a promising strategy for targeting advanced PCa, which may also be exploited as potential anti-cancer therapy for other cancer types.

## Results

### The transcriptional activity of FOXO1 is inhibited by PLK1-mediated phosphorylation in PCa cells

**Figure 1:**
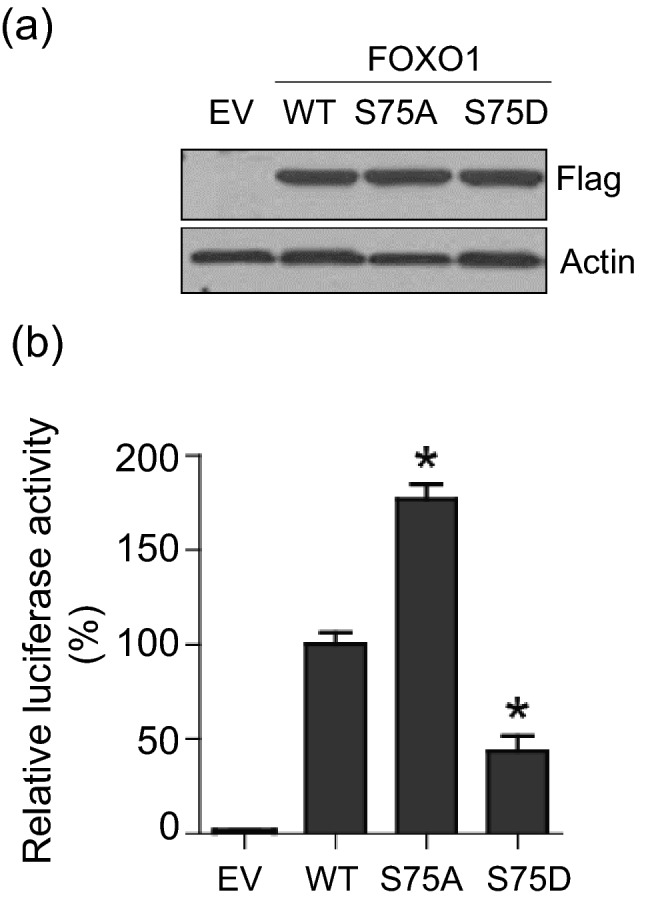
The transcriptional activity of FOXO1 is inhibited by PLK1-mediated phosphorylation in DU145 cells. (**a**) DU145 cells were transfected with plasmids encoding for either empty vector (EV), Flag-tagged FOXO1 WT, or a mutant (S75A or S75D). Exogenous FOXO1 expression was detected by western blot using anti-Flag antibody. (**b**) DU145 cells were transfected with a luciferase-based FOXO1 transcriptional activity reporter plasmid, a Renilla luciferase reporter and plasmids as indicated. Luciferase activities were measured 24 h after transfection. The experiment was repeated 3 times. (mean ± SD, *p < 0.05 vs. WT, Student's t-test).

We previously demonstrated that PLK1 phosphorylates FOXO1, which promotes the inhibition of FOXO1′s transcriptional activity in HeLa cells^18^. Using a luciferase-based FOXO1 transcriptional activity reporter plasmid, we investigated whether PLK1 phosphorylation of FOXO1 also causes the inhibition of FOXO1 transcriptional activity in PCa cells. In our previous report, we showed that Serine 75 is a major phosphorylation site and generated a series of FOXO1 mutants by mutating the PLK1 phosphorylation site to alanine (FOXO1-S75A) or aspartate (FOXO1-S75D) to either block or mimic PLK1 phosphorylation^18^. We thus examined the effects of these phosphor-mutants on FOXO1 transcriptional activity in 2 commonly used PCa cell lines, LNCaP and DU145. Compared to wild-type (WT) FOXO1, the phospho-resistant mutant FOXO1-S75A showed a significant increase in transcriptional activity in both cell lines (Figs. 1 and S1). In contrast, the phospho-mimicking mutant FOXO1-S75D exhibited a significant decreased in the FOXO1 transcriptional activity in both cell lines (Figs. 1 and S1). Consistent with our previous results in HeLa cells^18^, we found that PLK1-dependent phosphorylation of FOXO1 also has an inhibitory effect on FOXO1′s transcriptional activity in PCa cells.

### PLK1-mediated phosphorylation of FOXO1 leads to its nuclear exclusion in PCa cells

**Figure 2:**
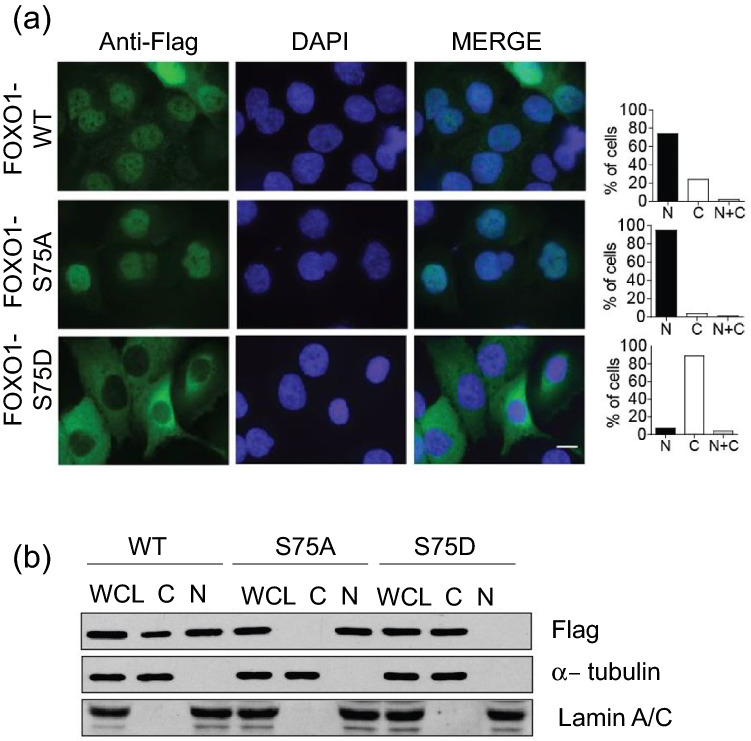
PLK1-mediated phosphorylation of FOXO1 leads to its nuclear exclusion in DU145 cells. (**a**) Representative images of the cellular localization of Flag-tagged FOXO1 WT and FOXO1 mutants (S75A and S75D) in DU145 cells immunostained for Flag (green) and Dapi (blue) (left). Quantification of a representative experiment is shown in the bar graph (right). Similar results were obtained from 3 independent experiments. Scale bar = 10 μm. (**b**) DU145 cells expressing either empty vector (EV), Flag-tagged FOXO1 WT, or a mutant (S75A or S75D) were also subjected to subcellular fractionation. The levels of exogenous FOXO1 in total (WCL), nuclear (N), and cytoplasmic (C) fractions were determined by Western blotting with anti-Flag antibody. Lamin A/C and α-tubulin were used as a nuclear marker and a cytoplasmic marker, respectively.

We previously demonstrated that PLK1 inhibits the transcriptional activity of FOXO1 by promoting its nuclear exclusion in U2OS cells^18^. We, therefore, investigated whether PLK1-dependent phosphorylation of FOXO1 also inhibits the transcriptional activity of FOXO1 via its nuclear exclusion in PCa cells. To do so, PCa cells (LNCaP and DU145) were transfected with plasmids encoding for either empty vector, Flag-tagged FOXO1 WT, or a phospho mutant (S75A or S75D), and analyzed the subcellular localization of the proteins by immunofluorescence using an anti-Flag antibody. Interestingly, 95% of the phospho-resistant mutant FOXO1-S75A was localized in the nucleus in DU145 cells, compared to 75% of FOXO1 WT. In contrast, the phospho-mimicking mutant FOXO1-S75D was mainly (90%) retained in the cytoplasm (Fig. 2a). In LNCaP cells, 63% of the phospho-resistant mutant FOXO1-S75A was localized in the nucleus compared to 15% of FOXO1 WT (Figure S2a). Subcellular fractionation assays in both PCa cell lines further confirmed the immunofluorescent results (Figs. 2b and S2b). These results suggest that PLK1-mediated phosphorylation of FOXO1 leads its nuclear exclusion, thereby impairing the nuclear transcriptional activity of FOXO1 in PCa cells. It has been reported by several groups that AKT is another upstream regulator of FOXO1. AKT-induced phosphorylation of FOXO1 leads to the export of FOXO1 from the nucleus^20^. Our previous studies have demonstrated that PLK1 regulates FOXO1 in an AKT-independent manner^18^. While DU145 cells have wild-type PTEN alleles, LNCaP cells harbor one deleted allele of PTEN and one mutated allele of PTEN and do not express PTEN protein. Due to dual inhibitory effects from PTEN loss and endogenous PLK1 in LNCaP cells, the majority of cells expressing FOXO1 WT were mainly retained in the cytoplasm (Fig. S2a). Given the fact that cells harboring phospho-mimicking mutant FOXO1-S75D show a significant reduction in their transcriptional activity when compared to those with FOXO1 WT (Fig. S1), it is tempting to speculate that additional regulatory mechanisms other than subcellular nuclear exclusion may also contribute to PLK1-mediated inhibition of FOXO1′s transcriptional activity, which warrants future investigation.

### The pro-apoptotic function of FOXO1 is restored by inhibiting PLK1-dependent phosphorylation of FOXO1 in PCa cells

**Figure 3:**
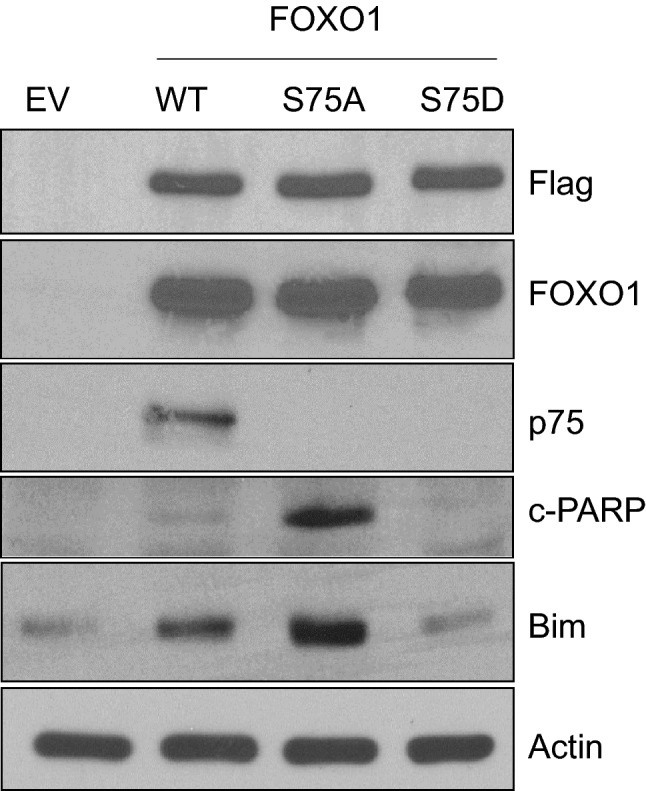
The pro-apoptotic function of FOXO1 is restored by blocking PLK1-dependent phosphorylation of FOXO1 in DU145 cells. DU145 cells were transfected with the indicated plasmids and harvested 72 h post-transfection. The cell lysates were subjected to western blot analysis with indicated antibodies.

FOXO1′s main function is to promote apoptosis by upregulating the expression of pro-apoptotic factors, such as Bim, FasL, NIP3, and legumain^4^. Thus, we explored whether PLK1-mediated phosphorylation of FOXO1 inhibits the pro-apoptotic activity of FOXO1 in PCa cells. We transiently transfected LNCaP and DU145 cells with Flag-tagged FOXO1 WT or a phosphorylation mutant and, 72 h post-transfection, analyzed the expression of Bim, a pro-apoptotic gene known to be a direct target gene of FOXO1^21^, by western blot. As expected, forced expression of FOXO1 WT or S75A induced the expression of Bim (Figs. 3 and S3). However, such upregulation was not observed in cells expressing the FOXO1-S75D mutant (Figs. 3 and S3). One of the most common signaling cascades involved in apoptosis is the activation of a highly specialized family of cysteinyl-aspartate proteases (caspases), which initiates cell death by cleaving several key proteins required for cellular functions and survival^22^. Poly (ADP-ribose) polymerase-1 (PARP-1) is such a cellular substrate of caspases, and is considered to be a hallmark of apoptosis^23^. We, therefore, analyzed PARP-1 cleavage as a readout of the caspases-dependent apoptosis induction. Overexpression of FOXO1 WT and S75A, but not the S75D mutant, in both PCa cell lines promoted the cleavage of PARP-1 (Figs. 3 and S3). Together, these data indicate that PLK1-mediated phosphorylation of FOXO1 inhibits the pro-apoptotic function of FOXO1 in a caspase-dependent manner.

### BI2536 and nocodazole synergistically impair the viability of advanced PCa cells

We showed that FOXO1′s pro-apoptotic activity in PCa cells was inhibited by PLK1-mediated phosphorylation (Fig. 3). Our previous studies demonstrate that these phosphorylation events mainly occur in the late phases of the cell cycle, at the time that PLK1′s activity is maximal. To explore this important finding, we tested whether FOXO1′s pro-apoptotic activity could be reactivated in the M phase of the cell cycle and exploited as a novel therapeutic strategy for advanced PCa. To this end, we combined nocodazole, a microtubule poison that arrests cells at M phase^24^, with a PLK1 inhibitor, leading to the reactivation of FOXO1′s apoptotic activity. Anti-cancer efficacy of such a combination therapy was tested on advanced PCa cells. Of note, microtubule-targeting chemotherapies are established treatments for metastatic PCa^25^, which further supports our hypothesis.

**Figure 4:**
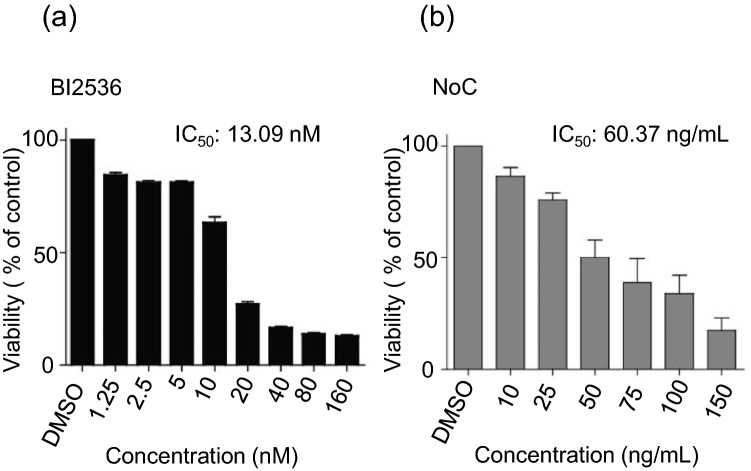
Effect of BI2536 and nocodazole treatment on viability of advanced PCa cells. (**a**) Dose–response curve of BI2536 treatment. Cell viability was assessed at 72 h of drug exposure using MTS assay. (**b**) Dose–response curve of nocodazole (NoC) treatment. Cell viability was assessed as in (**a**).

Several PLK1 inhibitors have been tested in phase I or II clinical studies in patients with various cancers, we used BI2536, which has been reported to be a potent and selective ATP-competitive PLK1 inhibitor^26^. We first determined the half maximal inhibitory concentration (IC_50_) value of BI2536 and nocodazole for DU145 cells, an aggressive PCa cell line. Cells were treated with various concentrations of BI2536 (0, 1.25, 2.5, 5, 10, 20, 40, 80, and 160 nM) or nocodazole (0, 10, 25, 50, 75, 100, and 150 ng/mL) for 72hrs, and an MTS assay was performed. The IC_50_ values of BI2536 and nocodazole in DU145 cells were 13 nM and 60 ng/mL, respectively (Fig. 4). However, approximately 15% and 20% of cells remain resistant to cell death upon treatment with BI2536 up to 160 nM or nocodazole up to 150 ng/mL, respectively (Fig. 4).

**Figure 5:**
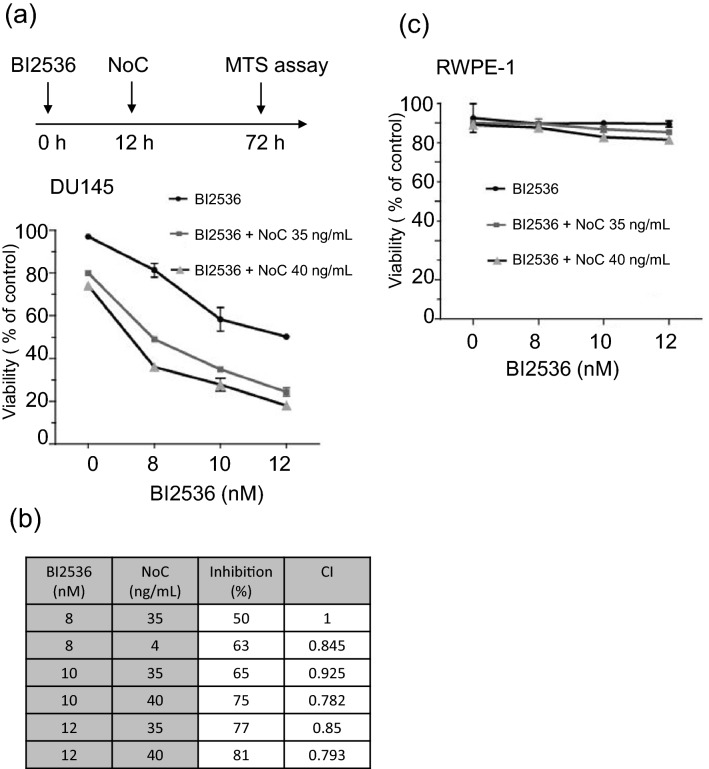
BI2536 and nocodazole synergistically impair the viability of advanced PCa cells. DU145 cells (**a**) and normal epithelial prostate RWPE-1 cells (**c**) and were treated for 72 h with BI2536 or NoC, alone or in combination of indicated concentrations. Cell viability was assessed as in Fig. 4. (**b**) Combination index (CI) for BI2536 and NoC treatment of DU145 cells were calculated using the Calcusyn program.

Clinical trials have revealed moderate side effects associated with high dose of BI2536 in monotherapy^27,28^. Reactivate the apoptotic function of FOXO1 at the late stages of the cell cycle may improve the anti-cancer efficacy of BI2536. Thus, we next evaluated whether combination of low dose of BI2536 and nocodazole enhances the cytotoxic effects in advanced PCa cells. We treated DU145 cells with various combinations of BI2536 (8, 10, and 12 nM) and nocodazole (35 and 40 ng/mL) (Fig. 5a). All of the dose levels evaluated were below IC_50_. Interestingly, all of the combinations exhibited a greater inhibitory effect on the viability of DU145 cells than BI2536 and nocodazole alone (Fig. 5a). To determine whether these cytotoxic effects are synergistic or additive, the combination indices (CIs) of the different combinations of nocodazole and BI2536 were determined using the CalcuSyn program^29^. For most of the dose combinations, the CI was below 1, which indicates that BI2536 and nocodazole act in a synergistic manner on PCa cell growth (Fig. 5b). We then tested whether the low doses of BI2536 and nocodazole used in combination therapy specifically induce cytotoxicity in advanced PCa cells, but not normal prostate epithelial cells. To this end, we treated RWPE-1, immortalized normal human prostate epithelial cells, with the same regimens as those used in DU145 cells. Importantly, nocodazole and BI2536, alone or in combination, had a slight, if any, adverse effect on the viability of RWPE-1 cells (Fig. 5c). These results strongly suggest that this combination treatment synergistically inhibits viability of advanced PCa cells, but not normal prostate epithelial cells.

### BI2536 in combination with nocodazole induces apoptosis in advanced PCa cells

**Figure 6:**
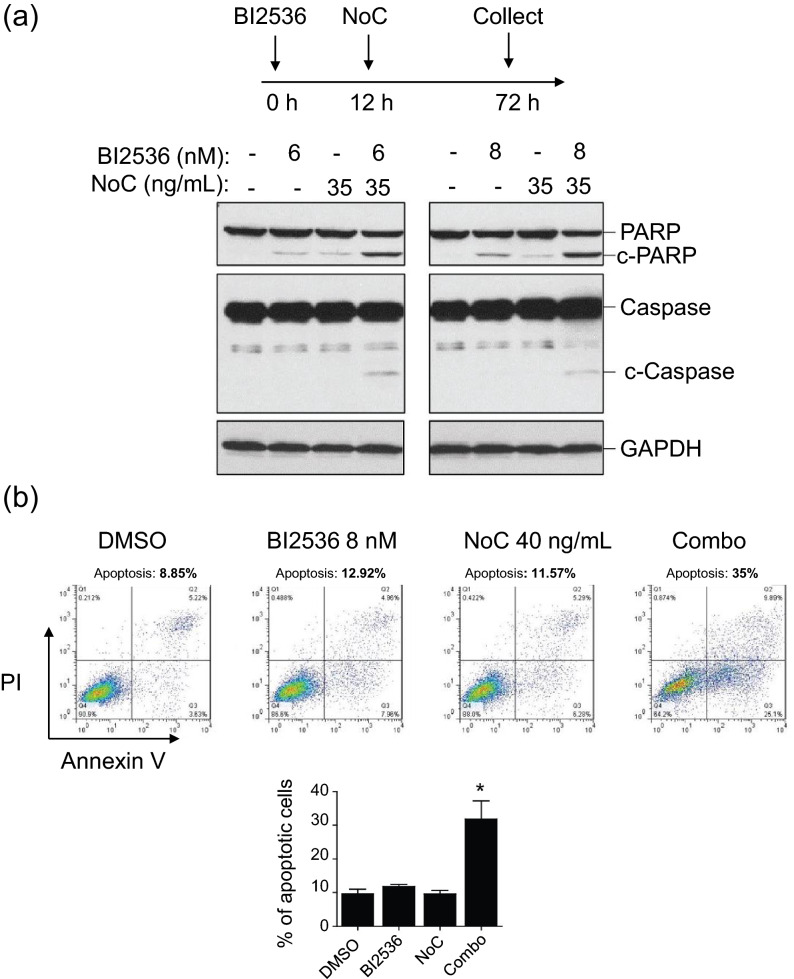
BI2536 in combination with nocodazole induces apoptosis in advanced PCa cells. (**a)** DU145 cells were treated with BI2536 and NoC for 72 h as indicated. Cells lysates were prepared and subjected to western blot anaysis with the indicated antibodies. GAPDH was used as an internal loading control. (**b**) DU145 cells were treated with BI2536 or NoC alone or in combination (combo). Cells were stained with Annexin V-FITC and PI 72 h post-treatment. Apoptotic cells were analyzed by flow cytometry. Graph represents the percentage of apoptotic cells as mean ± SD of 3 independent experiments.

Treatment of DU145 cells with BI2536 and nocodazole in combination showed significantly higher cytotoxicity than BI2536 and nocodazole individually (Fig. 5). To determine whether the synergistic growth inhibition was associated with efficient induction of apoptosis, hallmarks of the apoptotic cascade were examined. Consistent with the cell viability assays, cleaved PARP was significantly higher in DU145 cells treated with both nocodazole and BI2536 than in DU145 cells treated with nocodazole or BI2536 alone (Fig. 6a). We next assessed the externalization of phosphatidylserine (PS), as a hallmark of apoptosis, detected by Annexin V (+) using flow cytometry. The co-treatment caused approximately threefold increase in apoptosis compared to single treatment (Fig. 6b, *p* < *0.01*). While the nocodazole treatment led to a robust phosphorylation of FOXO1 by PLK1, the co-treatment caused significant reduction of FOXO1 phosphorylation as well as a strong induction in the expression of Bim, a downstream target of FOXO1, which further substantiates our working hypothesis and validates the mechanism of action of this novel combination therapy for advanced PCa (Figure S4). Collectively, these results indicate that the combination of BI2536 and nocodazole significantly induce apoptosis in advanced PCa cells.

**Figure 7:**
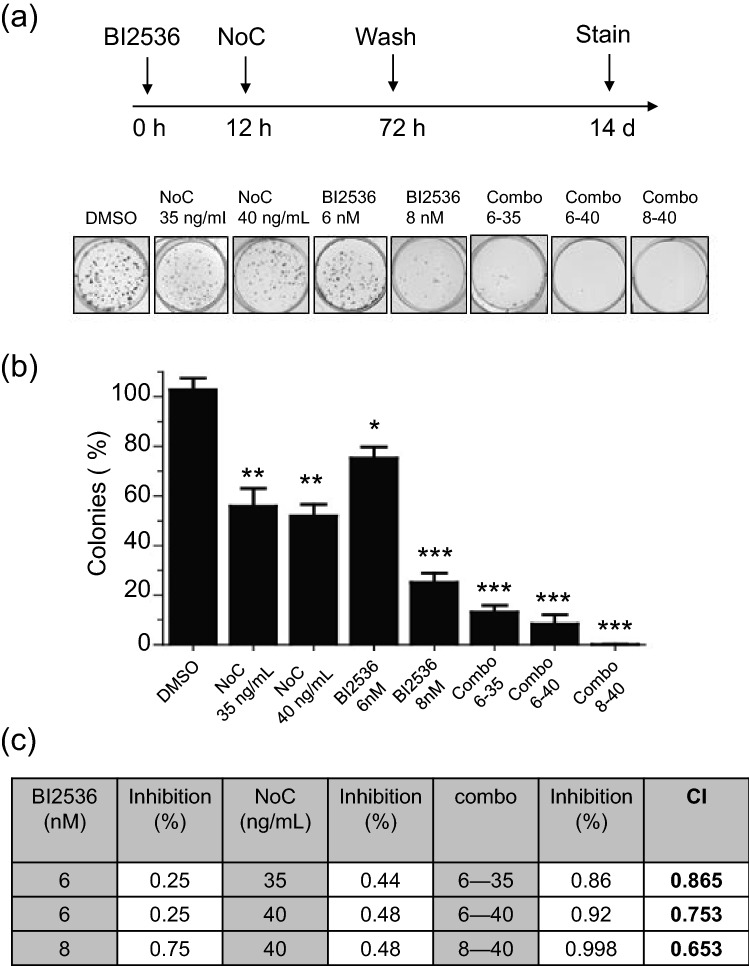
The combination of BI2536 and nocodazole efficiently diminishes the clonogenicity of advanced PCa cells. (**a**) Colony formation assays for the DU145 cell line treated with BI2536 or NoC alone or in combination (combo), or with DMSO (vehicle) for 10–14 days. (**b**) Quantified results were expressed as a percentage of the Control (DMSO) group, setting at 100%. Data are expressed as mean ± SD of three independent experiments (*p < 0.05; **p < 0.01, ***p < 0.001). (**c**) The combination index (CI) for BI2536 and NoC treatment of DU145 cells were calculated using the Calcusyn program.

We next analyzed the effect of short-term exposure to BI2536 and nocodazole alone or combination on long-term cell survival of advanced PCa cells using clonogenic assays. DU145 cells were treated with BI2536 [6 nM or 8 nM] and nocodazole [35 ng/mL or 40 ng/mL]) alone or in combination for 72 h, and clonogenic potential was assessed 14-days post-treatment. All the combinations exhibited a synergistic effect in DU145 cells (Fig. 7). Collectively, these results support that the inhibition of growth and survival of PCa cells by BI2536 can be markedly enhanced when used in combination with nocodazole.

## Discussion

Defects in apoptotic mechanisms play a crucial role in cancer development^30–32^. Drugs or treatment strategies that can restore defective apoptotic signaling pathways have the potential to improve the efficacy of cancer treatments. Thus, further deciphering the molecular mechanisms regulating apoptosis may provide a new therapeutic avenue for cancer treatment.

Several studies have shown that the activation of FOXO1 can induce apoptosis in cancer cells. For instance, it has been shown that the use of MHY-449, a novel cytotoxic drug, can induce apoptotic cell death by downregulating the phosphorylation levels of FOXO1 in PC3 cells^33^. Another study showed that Wortmannin can induce dephosphorylation and activation of FOXO1 via Bim signaling, which promotes apoptosis in non-Hodgkin lymphoma^34^. Moreover, restoration of FOXO1 activity contributes to a reduction in cancer cells due to a decrease in cell proliferation and induction of apoptosis in MCF-7 breast cancer cells^35^. Additionally, it has been shown that curcumin induces apoptosis in pancreatic cancer and glioma cells through the activation of FOXO1^36,37^.

The relevance of FOXO1 to human cancers is further evidenced by the fact that the tumor suppressor functions of FOXO1 are found to be disrupted by many oncogenic pathways^38^. Therefore, restoring the pro-apoptotic functions of FOXO1 in cancerous cells would be of great interest for the treatment of human cancers. In this study, we show that PLK1-mediated phosphorylation of FOXO1 induces its nuclear exclusion, leading to the inhibition of FOXO1′s nuclear transcriptional activity in PCa cells (Figs. 1 and 2). This suggests that the PLK1-FOXO1 pathway may be exploited as a new therapeutic strategy to reactivate the FOXO1-mediated apoptotic signaling networks and efficiently kill human advanced PCa cells.

Maximize therapeutic efficacy and minimize the therapy related toxicity of anticancer agents is an essential step in cancer treatment. Here, we have demonstrated that combining low dose of BI2536 with nocodazole (to synchronize cells in G_2_/M phase), to limit side effects, synergistically decrease cell growth and survival in advanced PCa cells (Figs. 5, 6, 7). Importantly, minimal effects on normal prostate epithelial cells were observed, highlighting the specificity of this strategy toward advanced PCa cells.

Expression of PLK1 is frequently elevated in various human cancers, including advanced PCa^39^. Importantly, high expression levels of PLK1 are correlated with poor clinical outcome^39^. These clinical findings suggest that increased PLK1 expression may be important for PCa progression. The results from this study support the hypothesis that aberrant expression of PLK1 leads to the inhibition of the tumor suppressor function of FOXO1, thereby contributing to PCa survival and/or resistance to therapy. The existence of PLK1-mediated FOXO1 inhibition in human advanced PCa cells and the effectiveness of inhibition of PLK1 in reversing this process highlight the therapeutic potential of targeting PLK1-FOXO1 signaling for the treatment of advanced PCa.

In conclusion, we provide evidence that PLK1-dependent phosphorylation of FOXO1 induces its nuclear exclusion and negatively regulates FOXO1 transcriptional activity in PCa. Inhibition of PLK1 in combination with nocodazole, resulting in reactivation of FOXO1-mediated apoptosis, synergistically inhibited cell growth and survival in advanced PCa cells. These findings shed light on a novel approach to reactivate apoptotic pathways in advanced PCa and support targeting PLK1-FOXO1 pathways as a novel therapeutic approach for treating advanced PCa.

## Materials and methods

### Prostate cancer cell lines

DU145, LNCaP and RWPE-1 cells (ATCC) were maintained and cultured at 37 °C in 5% CO2. DU145 and LNCaP were cultured in RPMI 1,640 medium (Thermo Fisher Scientific) supplemented with 10% (vol/vol) fetal bovine serum, 100 units/mL penicillin and 100 units/mL streptomycin. RWPE-1 cells were cultured in keratinocyte serum-free medium (KFSM) supplemented with bovine pituitary extract (BPE) and epidermal growth factor (EGF) (Thermo Fisher Scientific). BI2536 and nocodazole were purchased from Selleckchem and Sigma-Aldrich respectively.

### Luciferase assays

LNCaP and DU145 cells were transfected with luciferase-based FOXO1 transcriptional reporter and a *Renilla* luciferase reporter pRL-TK used as an internal control of luciferase activity. 24 h after transfection, the luciferase activity in cell lysates was measured using a dual luciferase kit (Promega). The promoter activity of FOXO1 WT was set at 100%, and relative luciferase activity is represented. Experiments were performed in triplicate.

### Immunofluorescence microscopy

DU145 cells grown on coverslips were fixed for 15 min in 4% paraformaldehyde solution and then permeabilized with 0.5% Triton X-100 for 5 min. Coverslips were blocked in 3% BSA for 1 h and then incubated with primary antibodies overnight at 4℃. After 3 washes with PBS-0.1% Triton X-100 cells for 5 min, coverslips were incubated with fluorescently conjugated Alexa Fluor 488 or 568 secondary antibodies (Life Technologies) for 45 min at room temperature. Cells were counterstained and mounted using Prolong gold mounting medium with DAPI (Molecular Probes). Images were taken using a Zeiss AxioImager A1 equipped with an Axiocam MRc color CCD camera and 63 × oil immersion lens. Analysis and quantification were performed using ImageJ software.

### Antibodies

Anti-FOXO1 (Cell Signaling Technology) and anti-Lamin A/C (Cell Signaling Technology) antibodies were used at dilutions of 1:1,000. Anti-α-tubulin (Sigma-Aldrich) antibody was used at a dilution of 1:5,000. Anti-β-actin (Sigma-Aldrich) and anti-GAPDH (Santa Cruz Biotechnology) antibodies were used at a dilution of 1:1,500. Anti-cleaved PARP (Santa Cruz Biotechnology), anti-Bim (Cell Signaling Technology), anti-Flag (Sigma-Aldrich) were used at dilutions of 1:2000. As described previously, rabbit polyclonal antibody recognizing phosphorylated serine 75 (anti-p75) of FOXO1 was generated by immunizing rabbits with the phosphorylated peptides DFMSNLpSLLEESEDC and affinity-purified using its corresponding peptide columns^18^.

### Subcellular protein fractionation

Subcellular protein fractionation was performed as previously described in^40^. Cells were lysed in hypotonic buffer (10 mM HEPES–KOH, 1.5 mM MgCl_2_, 10 mM KCl, 0.5 mM DTT, 0.2 mM PEFA 1,023, pH 7.9, 0.5% NP-40). The supernatants (cytoplasmic extracts) were collected after centrifugation for 10 s at 16,000 g at 4℃. After two washes with hypotonic buffer, pellets were lysed with high-salt buffer (450 mM NaCl, 1 mM PMSF, 50 mM Tris pH 7.4, 0.2 mM Na3VO4, 5 mM β-glycerophosphate, 20% glycerol, 2 mM DTT, 1% NP-40) for 10 min at 4℃ and centrifuged for 15 min at 16 000 g at 4℃. Supernatant were collected as nuclear extract.

### MTS assay

Cells were seeded in 96-well plates at a density of 2.5 × 10^3^ cells per well. 72 h after drug treatment, cell viabilities were measured by means of a CellTiter 96 AQueous One solution Cell Proliferation Assay (Promega) according to the manufacturer’s instructions. The experiments were performed I triplicate. The IC_50_ values were calculated from the average viability curves of 3 independent measurements for each condition using prism. The Combination Index of BI2536 and nocodazole were determined using the CalcuSyn program. CIs superior of 1 indicate an antagonism effect, of 1 indicate an additive effect, and inferior of 1 indicate synergetic effect.

### Measurement of apoptosis by Annexin V and PI staining

Cells were transiently transfected with indicated plasmids. At 48 h, cells were subjected to double staining for FITC-Annexin V and PI using detection kit (BD Biosciences) and analyzed by flow cytometry for apoptotic events according to the manufacturer’s instructions.

### Colony formation assay

DU145 cells were plated in 6-well plates in triplicate and cultured in medium containing BI2536 or nocodazole alone or in combination at different concentrations. Cells were incubated for 72 h before washing with PBS. Cells were cultured for an additional 14 days, fixed in 10% formalin and stained with 0.5% crystal violet. Colony numbers were counted in at least 3 independent experiments.

### Statistics

All experiments were performed at least 3 times in triplicates for each group. The results are presented as the mean ± SD. Statistical significance was determined using Student’s t-test, and the level of significance was set at *p* < 0.05.

## Supplementary information

Supplementary figures.
