# Supplementary material for: Targeting the PLK1-FOXO1 pathway as a novel therapeutic approach for treating advanced prostate cancer
Source: Sci Rep. 2020 Jul 23;10:12327. doi: 10.1038/s41598-020-69338-8 (PMC7378169; doi:10.1038/s41598-020-69338-8)
Supplement: Supplementary file 1 — Supplementary figures. [file 41598_2020_69338_MOESM1_ESM.pdf]

Targeting the PLK1-FOXO1 Pathway as a Novel Therapeutic Approach for Treating Advanced Prostate Cancer

Lilia Gheghiani[1], Shengzhe Shang[1], and Zheng Fu[1]*
[1]Department of Human and Molecular Genetics, VCU Institute of Molecular Medicine, VCU Massey Cancer Center, Virginia Commonwealth University, School of Medicine, Richmond, VA 23298, USA

*Correspondence: zheng.fu@vcuhealth.org

Supplemental figures

Figure S1

(a)

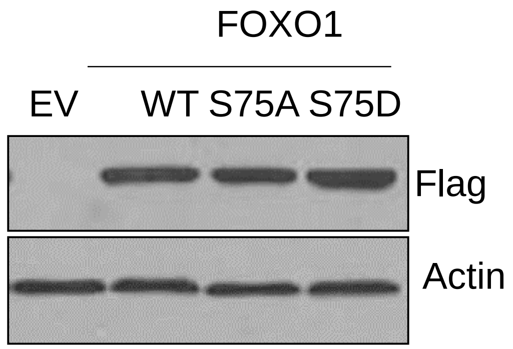

(b)

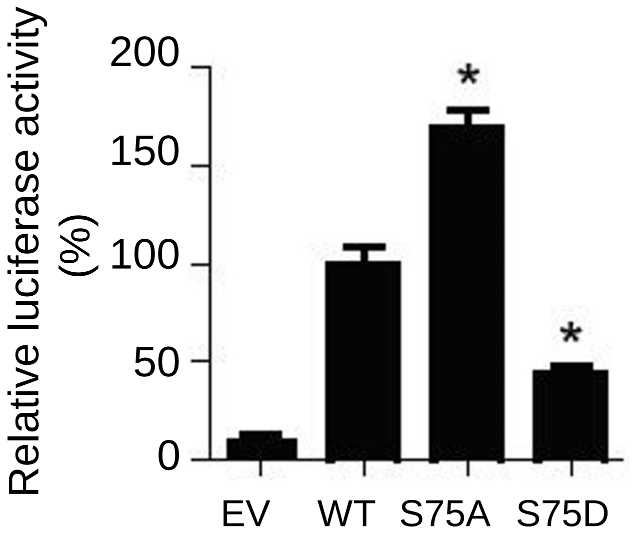

Figure S1. The transcriptional activity of FOXO1 is inhibited by PLK1-mediated phosphorylation in LNCaP cells. (a) LNCaP cells were transfected with plasmids encoding for either empty vector (EV), Flag-tagged FOXO1 WT, or a mutant (S75A or S75D). Exogenous FOXO1 expression was detected by western blot using anti-Flag antibody. (b) LNCaP cells were transfected with a luciferase-based FOXO1 transcriptional activity reporter plasmid, a Renilla luciferase reporter and plasmids as indicated. Luciferase activities were measured 24 hrs after transfection. The experiment was repeated 3 times. (mean ± SD, * $p < 0.05$ vs. WT, Student's t-test).

Figure S2

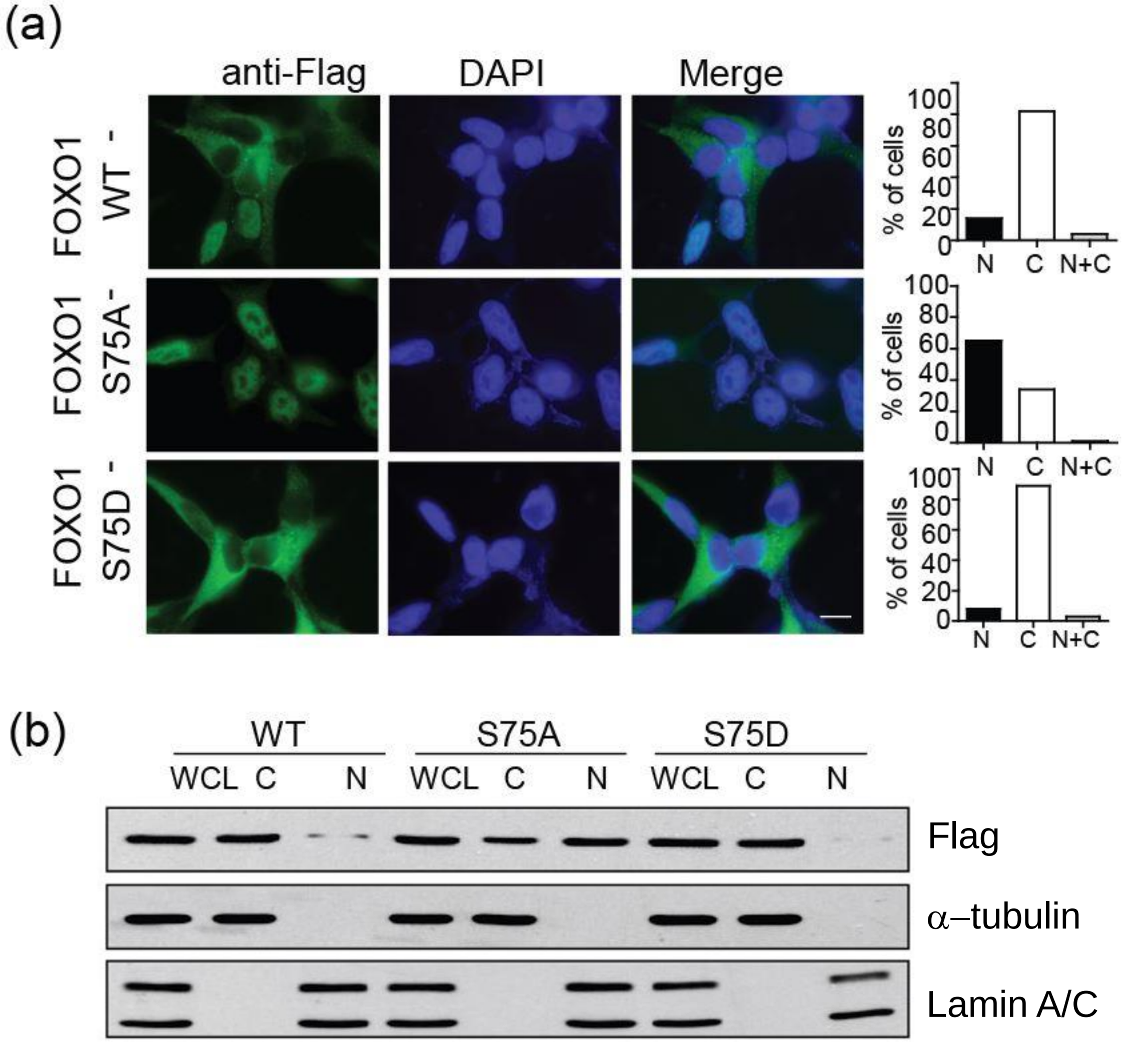

Figure S2. PLK1-mediated phosphorylation of FOXO1 leads to its nuclear exclusion in LNCAP cells. (a) The cellular localization of ectopically Flag-tagged FOXO1 WT and FOXO1 mutants (S75A and S75D) was examined by immunofluorescence staining using anti-Flag antibody. Quantification of a representative experiment is shown in the bar graph. Similar results were obtained from 3 independent experiments. Scale bar = 10 μm. (b) Cells as in (a) were subjected to subcellular fractionation. The levels of exogenous FOXO1 in total (WCL), nuclear (N), and cytoplasmic (C) fractions were determined by immunoblotting with anti-Flag antibody. The relative purity of the nuclear and cytoplasmic fractions was confirmed by sequential probing for the nuclear marker lamin A/C and the cytoplasmic marker α-tubulin, respectively.

Figure S3

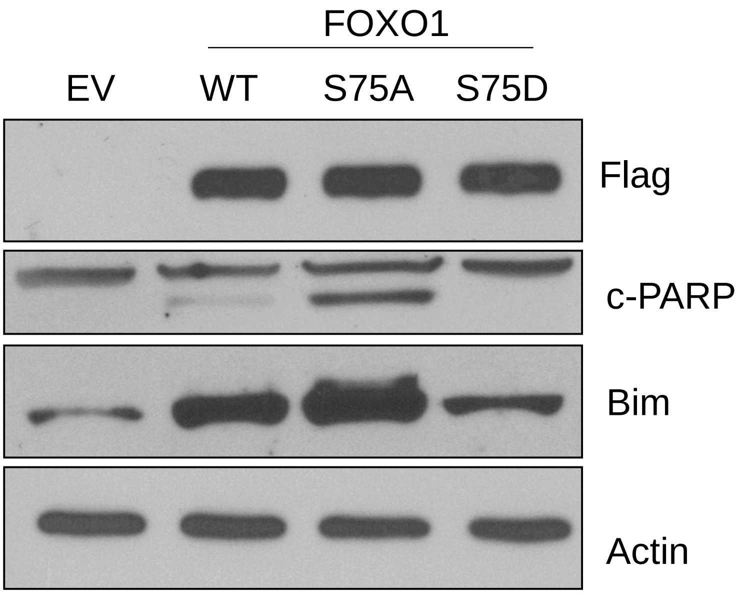

Figure S3. The pro-apoptotic function of FOXO1 is restored by blocking PLK1-dependent phosphorylation of FOXO1 in LNCAP cells. LNCaP cells were transfected with the indicated plasmids. Cells were harvested 72hrs post-transfection, and the expression of Bim, c-PARP, and FOXO1 was analyzed by western blot analysis. β-actin was used as loading control.

Figure S4

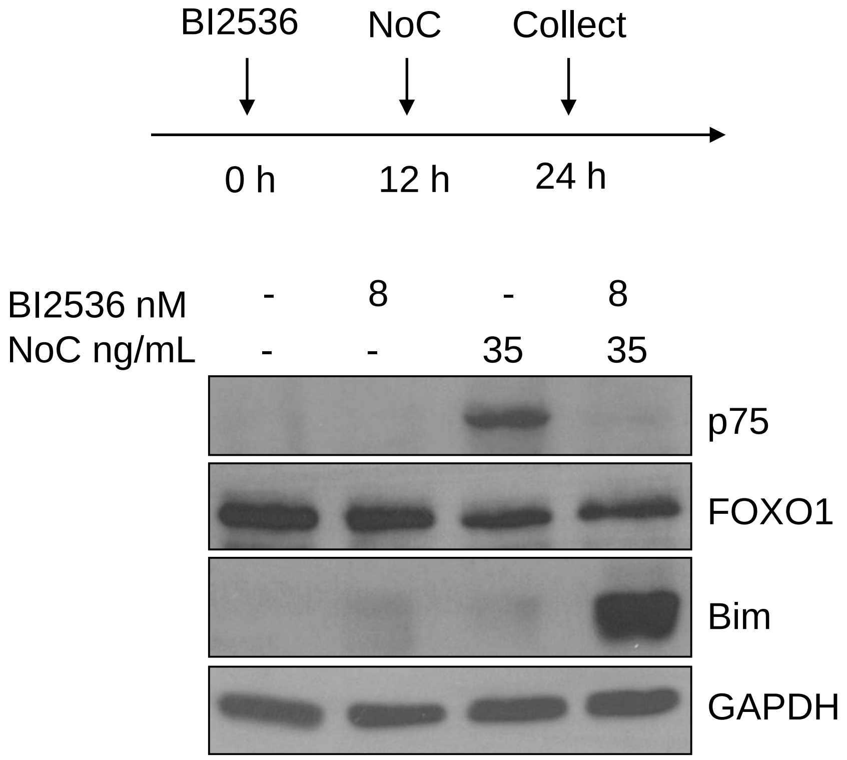

Figure S4. The co-treatment causes a reduction of FOXO1 phosphorylation and an induction of Bim expression in advanced PCa cells. DU145 cells were treated with BI2536 and NoC for 24 hrs as indicated. The cell lysates were subjected to western blot analysis with indicated antibodies.

Full Scan WB

Figure 1 and Figure S1

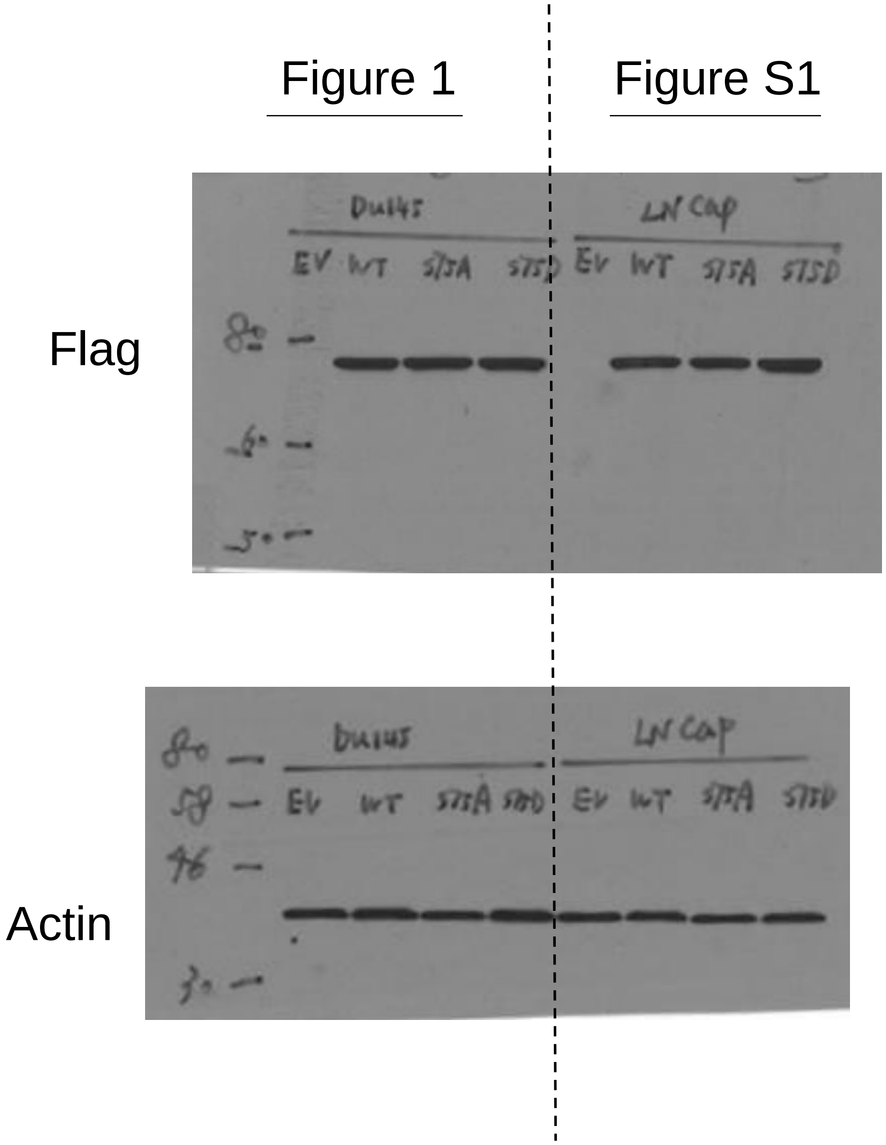

Figure 2

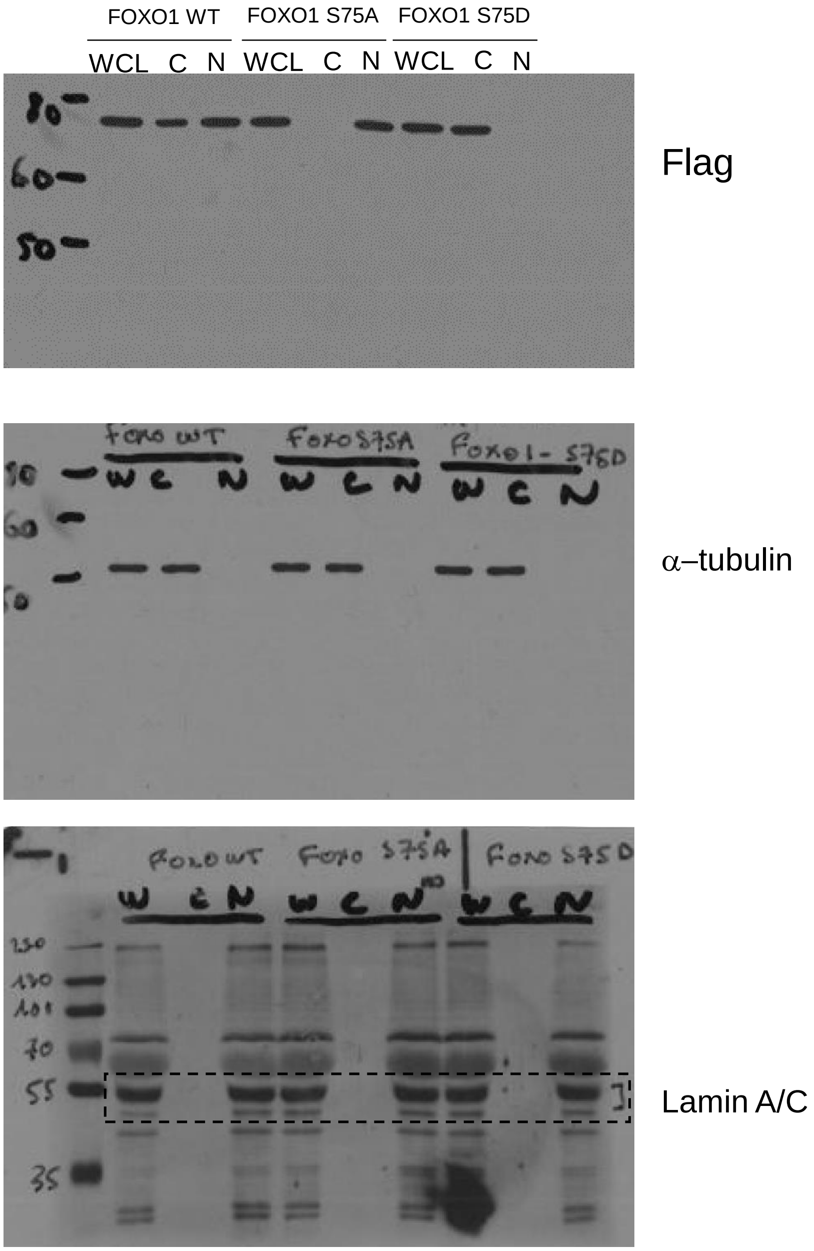
FOXO1 WT
FOXO1 S75A
FOXO1 S75D
WCL C N WCL C N WCL C N
Flag
α–tubulin
Lamin A/C

Figure 3

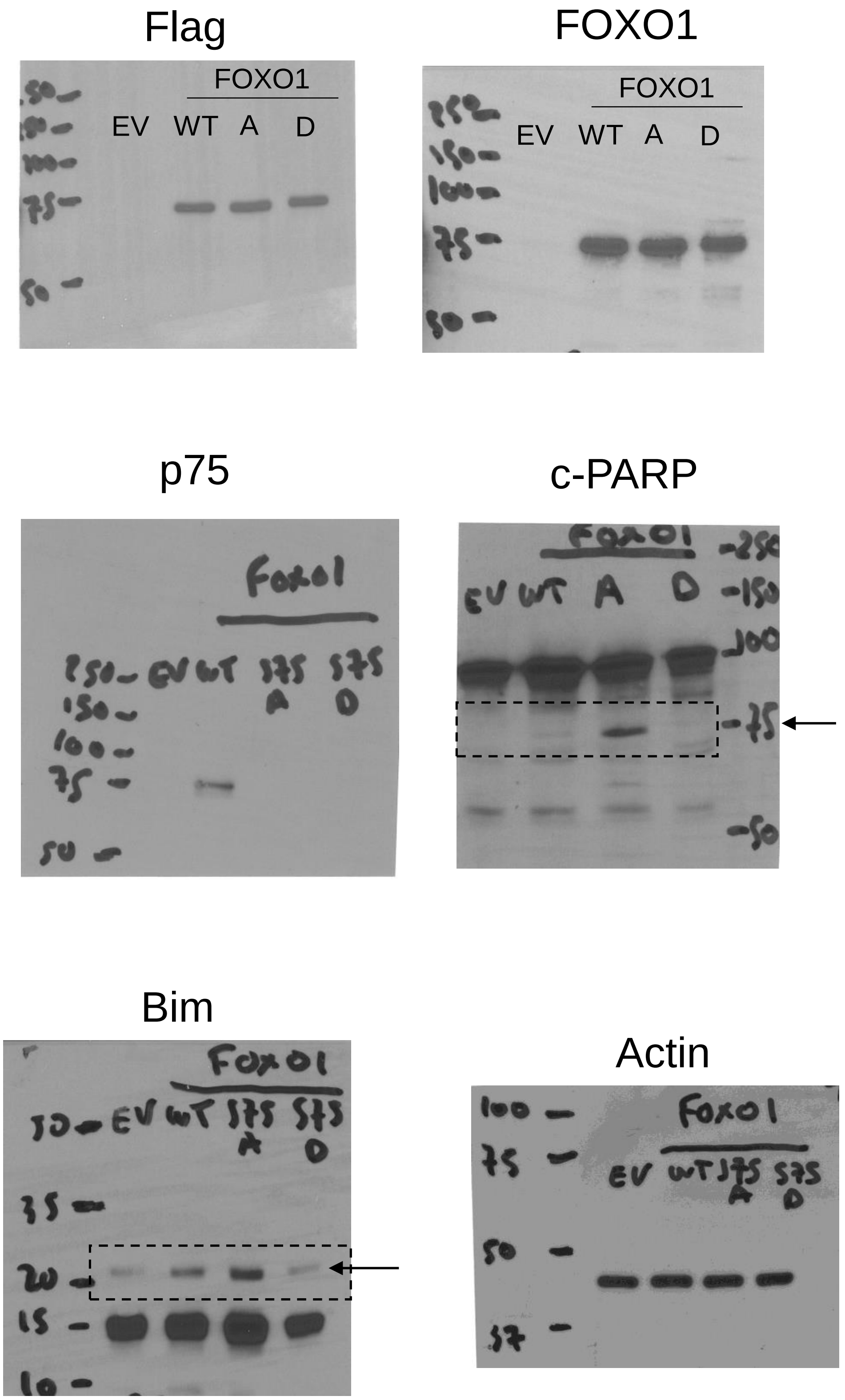

Figure 6

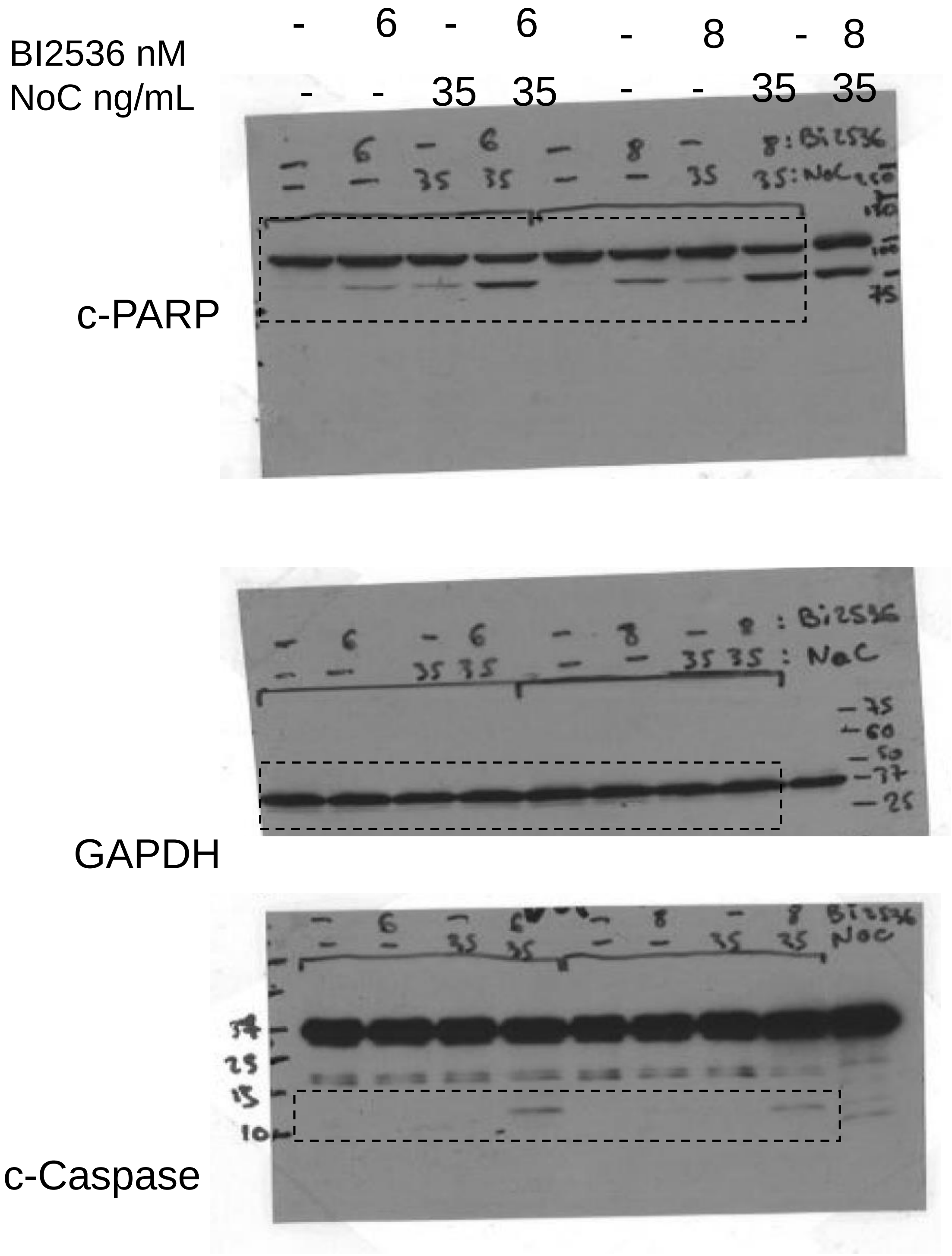

Figure S2

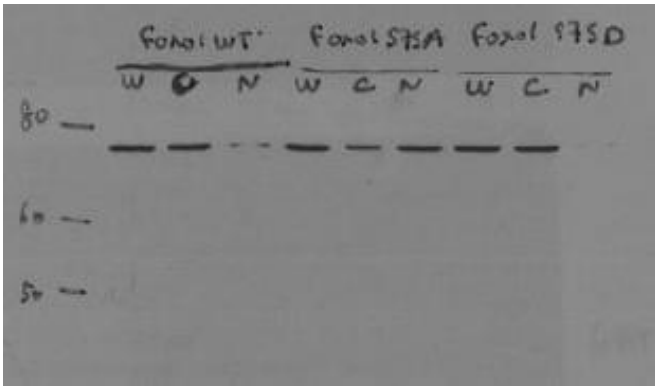

Flag

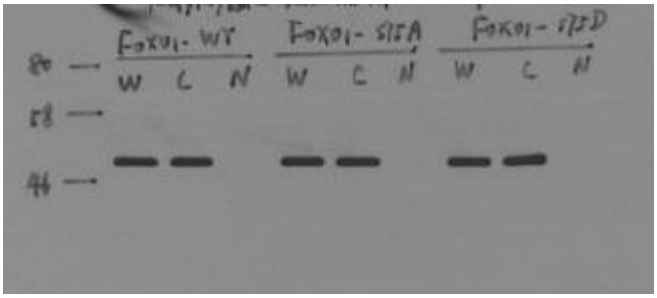

α–tubulin

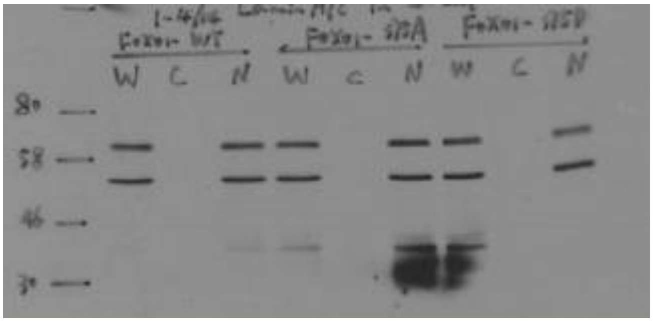

Lamin A/C

Figure S3

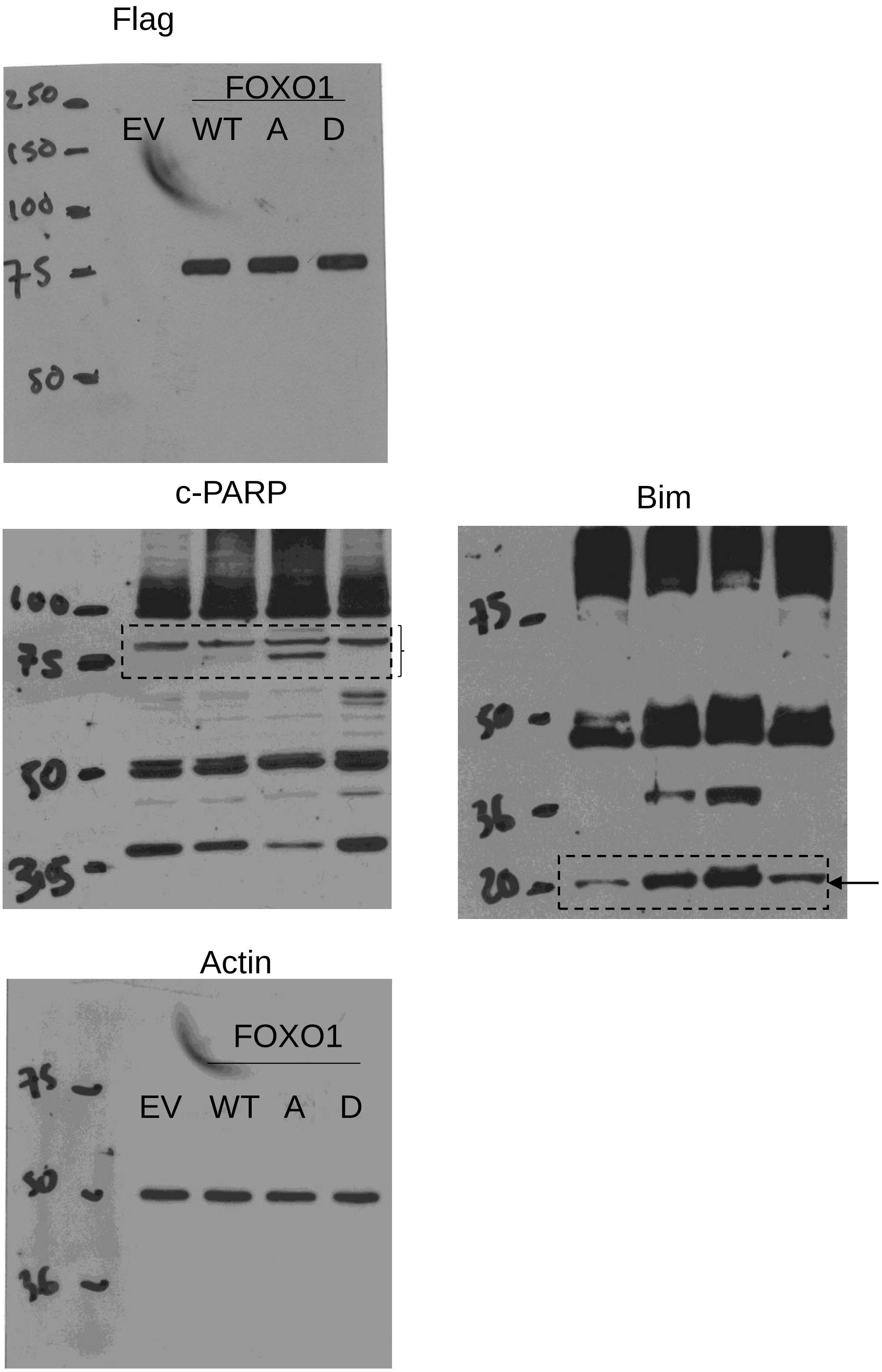
Flag
FOXO1
EV WT A D
250
150
100
75
50
c-PARP
100
75
50
35
Bim
75
50
36
20
Actin
FOXO1
EV WT A D
75
50
36

Figure S4

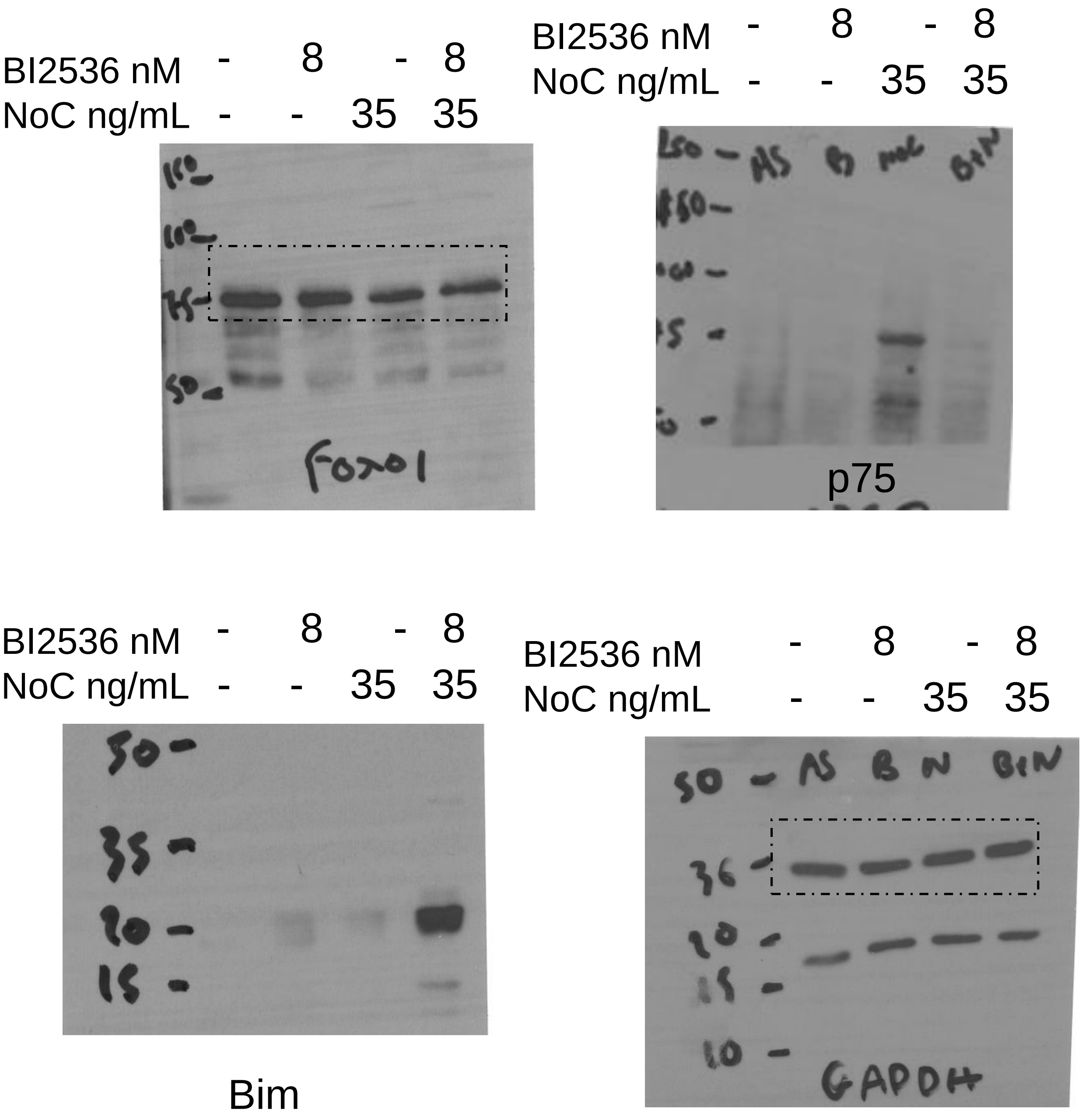
BI2536 nM - 8 - 8
NoC ng/mL - - 35 35
Foxo1
BI2536 nM - 8 - 8
NoC ng/mL - - 35 35
p75
BI2536 nM - 8 - 8
NoC ng/mL - - 35 35
Bim
BI2536 nM - 8 - 8
NoC ng/mL - - 35 35
GAPDH
